# Chromatin organization and global regulation of *Hox* gene clusters

**DOI:** 10.1098/rstb.2012.0367

**Published:** 2013-06-19

**Authors:** Thomas Montavon, Denis Duboule

**Affiliations:** 1 National Research Centre ‘Frontiers in Genetics’, School of Life Sciences, Ecole Polytechnique Fédérale, Lausanne, Switzerland; 2 National Research Centre ‘Frontiers in Genetics’, Department of Genetics and Evolution, University of Geneva, Geneva, Switzerland

**Keywords:** embryonic patterning, collinearity, chromatin architecture, long-range regulation, nuclear organization

## Abstract

During development, a properly coordinated expression of *Hox* genes, within their different genomic clusters is critical for patterning the body plans of many animals with a bilateral symmetry. The fascinating correspondence between the topological organization of *Hox* clusters and their transcriptional activation in space and time has served as a paradigm for understanding the relationships between genome structure and function. Here, we review some recent observations, which revealed highly dynamic changes in the structure of chromatin at *Hox* clusters, in parallel with their activation during embryonic development. We discuss the relevance of these findings for our understanding of large-scale gene regulation.

## 1. Introduction

Understanding the relationship between the structural organization of genomes, on the one hand, and their transcription during development, ageing and pathogenesis, on the other hand, is a major challenge of the post genomic-sequencing era. In recent years, considerable efforts have been devoted to the identification of functional elements within non-coding genomic intervals, as well as to the mapping of chromatin structure and spatial organization of chromosomes in the nuclear space [1–3]. These large-scale approaches tentatively define global relationships between the chromatin structure observed at a particular genomic locus, the three-dimensional organization of this locus and the associated gene activity. For instance, both local chromatin post-translational modifications as well as long-range physical interactions are used to identify candidate transcriptional regulatory elements and their target genes, genome-wide.

The emerging picture suggests that genes playing particularly important roles during embryonic development generally display a highly intricate regulatory organization locally, which involves a large number of control elements. Such elements are usually dispersed within large flanking regions which can be several hundreds of kilobases long and they establish complex patterns of contacts with their target genes. In a broader genomic context, active and inactive gene loci seem to occupy distinct nuclear spaces, which may relate to specialized nuclear compartments such as transcription factories [4–6]. These novel approaches are nevertheless largely descriptive and the future integration of these large datasets within a functional framework, for example by combining them with genetic approaches, will be critical to firmly establish the physiological relevance of these structural parameters.

The coordinated transcription of *Hox* genes within their respective genomic clusters has been a paradigm to study these questions, ever since high-throughput technologies have been available. This is mostly due to the structure of these loci, which display one of the highest concentrations of genes in the genome (in average ten genes within 120 kb) and their enigmatic transcriptional regulation, in response to both local and long-range strategies. Because these genes have a tightly constrained topological organization, they often have been (and still are) used as examples to illustrate the relationships between genome structure and function. Here, we review recent progress in deciphering the potential mechanisms, which may link chromatin organization to the transcriptional control of *Hox* genes *in embryo*.

## 2. Structural and functional organization of *Hox* clusters

*Hox* genes encode homeodomain transcription factors critical for the proper establishment of regional identities along the main body axis of bilaterian animals. In many species, they are grouped into genomic clusters, which share a conserved structural organization, indicating that a clustered set of genes was probably present in the ancestor of all extant bilateria. This ancestral cluster was split in the *Drosophila* lineage, where eight *Hox* genes are distributed in the *Antennapedia* (*Ant-C*) and *Bithorax* (*BX-C*) complexes; in contrast, the two rounds of whole-genome duplications that accompanied the emergence of vertebrates led to the presence of four paralogous *Hox* clusters in this group, termed *HoxA* to *HoxD*, where a total of 39 genes can be scored in mammals (see [7] for references).

This peculiar genomic organization is closely associated with a regulatory process referred to as ‘collinearity’, i.e. tight correspondence that exists between the order of *Hox* genes within each cluster, on the one hand, and the succession of their expression territories along the anterior–posterior embryonic axis, on the other hand. This property, referred to as spatial collinearity, was originally proposed by studying the genetics of the *Drosophila* BX-C [8]. It was subsequently extended to vertebrates [9–11], which indicated that animals apparently displaying highly divergent morphologies nevertheless rely on the same genetic systems to pattern their body plans. Part of this spatial collinear distribution of *Hox* expression domain is caused by a temporal sequence in their transcriptional activation, which reflects their genomic order, along with the extension of the embryonic axis (‘temporal collinearity’ [12]). A tight control of *Hox* genes transcription, in both space and time, is thus critical for proper development, because variations in the expected HOX proteins combinations at any given anterior–posterior level in the embryo usually lead to alterations in axial patterning [13,14].

Because of this capacity to initiate a coordinated transcriptional response of this series of genes involved in patterning processes, these collinear properties were co-opted several times in the course of vertebrate evolution, along with the emergence of structures displaying some kind of axial specification, such as the gut or the appendages. In such cases, specific *Hox* gene clusters were recruited, usually via the evolution of global regulation involving remote control elements, leading to the global transcription of several *Hox* genes at once ([15]; see below). The mechanistic relationship(s) between *Hox* genes clustering and collinear regulation, however, is still elusive. For example, in some animal species, *Hox* genes display this peculiar expression patterns along the embryonic axis, even though the clustered organization has been lost ([16]; see [7] for a discussion of this issue). Furthermore, *Hox* genes often, yet not always, recapitulate some aspects of their genuine expression specificities in the developing trunk, when isolated from their endogenous cluster and integrated at random genomic locations as transgenes, indicating that transcription units carry some of the requested regulatory information [17,18].

Despite these observations, targeted modifications of *Hox* clusters have shown that changing the relative position of a gene within its cluster has a critical impact on several aspects of its transcriptional regulation, such as the timing of expression along the main axis, as well as transcript distribution along secondary axes, such as the growing limbs [18–22]. Therefore, global regulatory influences act over *Hox* clusters as a whole, on the top of more local, gene-specific proximal controls. A transition in chromatin structure, from an initially repressed state to a configuration progressively open for transcription, was proposed to accompany this coordinated activation early on [23]. We discuss below recent observations, which suggest that both local histone modifications and changes in the higher-order organization of chromatin are involved in the transcriptional control of *Hox* clusters.

## 3. Epigenetic control by Polycomb and Trithorax complexes

The epigenetic regulation of *Hox* gene clusters seems to rely, mostly, on the activities of protein complexes encoded by *Polycomb* (*PcG*) and *Trithorax* (*TrxG*) group genes. During early *Drosophila* embryogenesis, maternally supplied transcription factors define the spatial patterns of *Hox* genes' activity [24]. These patterns are maintained at later developmental stages through the action of *PcG* genes, required for stable *Hox* gene repression. *TrxG* genes, on the other hand, counteract PcG silencing and keep *Hox* genes expressed in the appropriate domains [25].

PcG and TrxG gene products are found in multi-protein complexes, which mediate the post-translational modification of histone tails and thus affect chromatin structure. PcG-mediated silencing relies on the combined actions of the Polycomb repressive complexes 1 and 2 (PRC1 and PRC2). PRC2 tri-methylates lysine 27 of histone H3 tail (H3K27), a mark that is tightly associated with gene repression [26–29]. PRC1 is recruited to H3K27me3 [26,29–31] and contains the Ring1B E3 ubiquitin ligase that triggers the ubiquitylation of H2A at lysine 119 [32,33]. By contrast, TrxG complexes are responsible for the tri-methylation of histone H3 tail at lysine 4 (H3K4me3), a chromatin mark associated with transcriptional activation [34].

In mouse and human cultured cells, H3K27me3 and H3K4me3 predominantly decorate silent or active promoters, respectively [35–38]. Surprisingly, a significant number of silent loci, including *Hox* clusters, display both H3K4 and H3K27 tri-methylation in pluripotent embryonic stem (ES) cells, a chromatin signature referred to as ‘bivalent domains’ [39,40]. Differentiation of ES cells leads to a resolution of these bivalency into either active or repressive chromatin marks, suggesting that such bivalent domains might label genes that are kept silent, but ‘poised’ for rapid activation upon lineage commitment. Such plasticity is at odds with the classical view of PcG and TrxG function as mediators of a stable memory of epigenetic states [41], but is supported by the identification of H3K27 lysine de-methylases recruited to gene promoters upon transcriptional activation [42–45].

**Figure 1.**
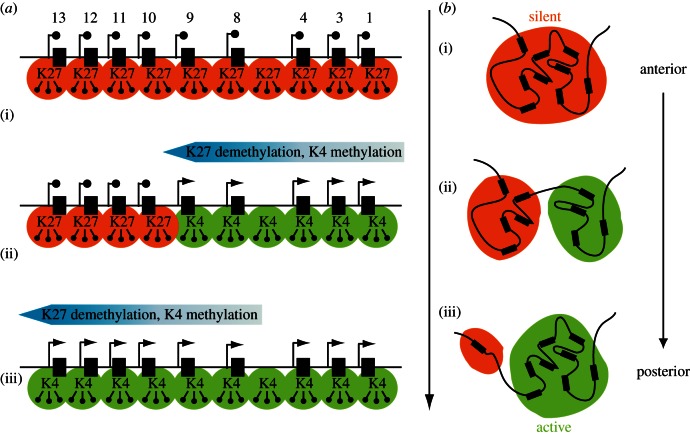
Collinearity during trunk extension and chromatin dynamics at *Hox* clusters. Expression of *Hox* gene along the anterior-to-posterior (AP) embryonic axis is collinear with gene order within the cluster. (*a*) During axial extension, the sequential onset of *Hox* gene transcriptional activation is accompanied by a transition in histone modifications over the gene cluster. In ES cells (i), the whole cluster is labelled with H3K27me3 (orange), a mark associated with Polycomb-mediated silencing. In the developing embryo, this mark is progressively erased and replaced by H3K4me3 (green), concomitantly with gene activation. (*b*) Active and silent *Hox* loci segregate into distinct spatial compartments along the AP axis. In embryonic tissues where the whole cluster is repressed, such as the forebrain (i), *Hox* clusters form a compact three-dimensional structure. In regions where subsets of *Hox* genes are expressed (anterior trunk, ii), active and silent genes segregate in distinct compartments, labelled with either H3K27me3 (silent compartment) or H3K4me3 (active compartment). In posterior embryonic regions (iii), most genes are transcribed and participate in the active compartment.

Similar to their *Drosophila* orthologues, *PcG* and *trxG* genes are required for vertebrate embryonic development, and mutations in these genes lead to a deregulation of *Hox* genes, amongst other defects (reviewed in [46,47]). Recently, a progressive loss of H3K27me3, concomitantly to a gain of H3K4me3, was observed together with the sequential activation of *Hoxd* genes during the extension of the main body axis in the mouse *in vivo* ([48]; figure 1*a*). Transcriptional activation of *Hoxd* genes thus occurs within the region of transition between these two epigenetic states, in a window that shifts along with time, from one extremity of the cluster to the other. The analysis of embryos carrying an engineered split *HoxD* cluster further revealed that clustering is not necessary for the initial deposition of H3K27me3, yet it is required for a fully coordinated transition in histone modifications [48].

The mechanisms recruiting PcG proteins to their target loci are not fully understood. In *Drosophila*, relatively short sequences termed Polycomb response elements (PREs) seem to be both necessary and sufficient for PcG recruitment and gene silencing, although these sites cannot be defined by a consensus DNA sequence [25]. The situation is more complex in vertebrates, where classical PREs—as defined in flies—remain to be identified. For instance, an element from the human *HOXD* cluster was recently reported to bind PcG proteins and to induce repression of a reporter gene [49]. However, the deletion of the murine orthologous sequence did not cause any dramatic change in *Hoxd* gene regulation [50], suggesting that it is not critical for PcG recruitment or, alternatively, that it is part of a robust mechanism with high compensatory capacities. Interestingly, CG-rich sequences, which are particularly abundant within *Hox* clusters, were proposed as a hallmark of PcG target promoters, at least in ES cells [51,52]. Silencing at these particular loci might thus involve the cooperative activity of multiple DNA elements.

## 4. Higher-order chromatin organization

The coordinated control of these dynamic epigenetic states might be facilitated by the spatial compartmentalization of *Hox* clusters. Changes in higher-order chromatin organization of *Hox* clusters were first observed by microscopy approaches such as fluorescent *in situ* hybridization (FISH), which allow the visualization of specific loci within the nucleus. Differentiation of ES cells after retinoic acid treatment leads to a decompaction of the *HoxB* cluster that parallels its global transcriptional activation [53]. Furthermore, *Hox* clusters are also less compact in those embryonic territories where *Hox* genes are probably active, than in silent regions [54]. However, the resolution of this approach did not allow the stepwise, collinear transition to be documented.

Also, owing to its limited resolution, FISH cannot currently provide a precise mapping of the three-dimensional organization of *Hox* loci, in either their active or inactive states. The development of the chromosome conformation capture (3C) technique, which provides an estimation of the average frequency of specific DNA–DNA ‘contacts’ (or proximity, see below) within a cell population, has greatly helped to overcome this limitation. While early 3C analyses only addressed interactions between a limited number of sequences, variant approaches such as 4C (circular 3C), 5C (3C carbon copy) or Hi-C generate rather unbiased datasets, where the mapping of all sequences contacting a locus of interest, or the analysis of mutual interactions between a large number of pre-determined sites—or even within an entire genome, can be produced [55]. Using these approaches, changes in the three-dimensional organization of *Hox* clusters were detected upon cell differentiation in culture or between cell lines derived from various regions of the body [56–58].

In dissected murine embryonic tissues, this configuration is tightly associated with the transcriptional activity of the gene clusters. In tissues were *Hox* genes are silent, such as the developing forebrain, *Hox* gene clusters form distinct spatial structures, as defined by widespread interactions between the various gene loci, within each cluster. In contrast, in regions where distinct subsets of *Hox* genes are transcribed, active and repressed genes segregate into distinct compartments, labelled by different chromatin marks [59,60]. In anterior regions of the main embryonic axis, expressed *Hox* genes appear to cluster together within a H3K4me3 decorated domain, whereas silent genes form a distinct compartment marked with H3K27me3. In more posterior areas, most *Hox* genes are found in the active compartment, suggesting a dynamic reorganization of the three-dimensional micro-architecture of these clusters ([59]; figure 1*b*).

This spatial separation between the active and silent parts of *Hox* clusters might in part reflect the PcG-mediated compaction of repressed loci, as shown *in vitro* [61]. Interestingly, the loss of either *Eed* (a protein member of PRC2) or *Ring1B* (a protein member of PRC1) leads to the decompaction of *Hox* clusters in ES cells [62]. In *Drosophila*, Polycomb targets segregate into discrete nuclear foci termed Polycomb bodies [63]. Despite a genomic distance of nearly 10 megabases (Mb), the *Ant-C* and *BX-C* complexes contact each other in a PcG dependent manner, in embryonic regions where both loci are silent [64].

Whether these patterns of contacts participate in the mechanism controlling *Hox* gene collinearity or, instead, merely reflect a general tendency of different chromatin segments to segregate into distinct nuclear domains depending on their modifications, remains to be established. However, the presence of separate spatial domains, as seen by using 4C, makes a biochemical artefact induced by cross-linking unlikely. In such a case, chromatin domains labelled either by H3K27me3 or by H3K4me3 would indeed be expected to cross-link together. Instead, a physical separation between active and repressed *Hox* subsets might participate in a tighter control of the sequential activation of *Hox* clusters, by isolating posterior genes from early/anterior activating influences.

## 5. Long-range control and regulatory archipelagos

The type of collinear regulation described above follows a mechanism acting purely in *cis*, and is shared by all paralogous *Hox* clusters. Therefore, it may represent an ancestral mechanism at work in those animals that activate their *Hox* genes in a precise time sequence (see above). In addition, in the vertebrate lineage, specific *Hox* clusters have evolved additional patterning functions [23,65,66], which required the emergence of novel regulatory modalities. These novel regulatory specificities often rely upon enhancer elements located outside the gene clusters, at a distance, likely to prevent deleterious effects of evolving additional regulatory elements within the gene clusters themselves, where the *cis-*regulatory sequences necessary to implement the ancestral collinear regulation along the anterior–posterior axis are mostly located [67].

Such potent enhancers have indeed been described and can act over large distances. They sometimes also affect unrelated, bystander transcription units, thus defining the concept of genomic ‘regulatory landscapes’ [68]. The emergence of these long-range controls, acting on several neighbouring *Hox* genes in a coordinated manner may have provided a selective pressure for the gene clusters to consolidate their structural organization in vertebrates [7].

**Figure 2.**
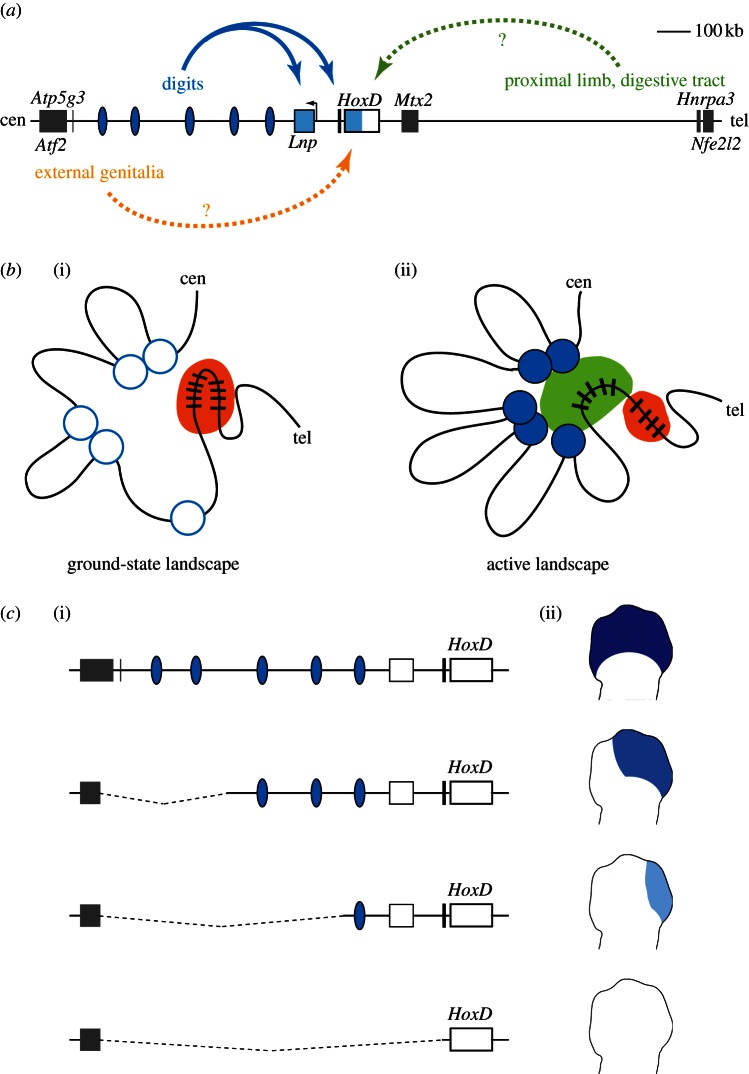
Long-range control and regulatory archipelagos. The coordinated transcription of *Hox* genes in different embryonic territories relies on remote regulatory elements located on either sides of the cluster. (*a*) Map of the *HoxD* locus and its flanking centromeric (cen) and telomeric (tel) conserved gene deserts. Multiple regulatory islands (blue ovals) participate in *Hoxd13–Hoxd10* regulation in developing digits (blue arrow). *Hoxd* gene activation in other embryonic structures also relies on long-range controls, yet the corresponding regulatory elements have not yet been identified (dashed arrows). (*b*) Spatial conformation of the locus. (i) In the silent state, a ground-state structure is formed, involving contacts between a subset of the regulatory elements and the *HoxD* cluster. (ii) In digits, additional contacts are formed, leading to a fully active conformation paralleled with histone modifications at the regulatory elements, and leading to *Hox* genes transcriptional activation. (*c*) Robustness of regulatory archipelagos. Different genetic configurations of the locus are shown in (i), with a scheme of the resulting *Hox* gene expression in developing limbs in (ii). The wild-type situation is depicted on top, and serial deletions within the archipelago are indicated below. Deleting subsets of the regulatory islands leads to partial downregulation of *Hoxd* genes in distal limbs, and only a full deletion of the regulatory interval fully abolishes transcription.

The transcription of *Hoxd* genes in developing limbs is the clearest example of such kind of acquired distal regulations around *Hox* gene clusters, as it has been investigated in some detail using both genetic and biochemical approaches. ‘Posterior’ *Hoxd* genes (from *Hoxd9* to *Hoxd13*) are required for the patterning of both proximal (arm and forearm or leg and lower leg) and distal (hands and feet) limb segments [69]. Their expression in growing limb buds follows two independent phases [21,70], controlled by distinct regulatory elements located on either side of the gene cluster [71]. In a first phase, corresponding to the future proximal limb segment, *Hoxd* genes are activated in a time sequence, which generates a nested pattern of expression along the limb anterior to posterior axis. While regulatory elements controlling this early activation are not yet reported, some genomic rearrangements suggest that they may be located on the telomeric side of the gene cluster, on chromosome 2 ([68,71]; figure 2*a*). Multiple highly conserved non-coding DNA sequences can be found there and thus represent potential candidates [72].

Subsequently, the transcription of posterior *Hoxd* genes (*Hoxd13* to *Hoxd10*) is activated at the distal extremities of the growing limbs, in a territory corresponding to presumptive digits. This ‘late phase’ is controlled by elements located centromeric from *Hoxd13* [73]. The mapping of the three-dimensional organization of the cluster revealed that active genes establish numerous long-range interactions with sequences dispersed over an 800 kb interval overlapping a gene desert located centromeric to the cluster [60]; figure 2*a*). These sites are grouped into ‘islands’ that are labelled with histone marks usually associated with enhancer elements, such as the monomethylation of H3K4 (H3K4me1) and the acetylation of H3K27 (H3K27Ac) [74–76]. The various islands contact each other, as well as the active part of the *HoxD* cluster, suggesting that they form an active conformation in developing digits.

In contrast, silent genes display the opposite profile of interactions, mostly involving the telomeric gene desert, which is devoid of active histone marks [60]. Therefore, the compartmentalization of *Hox* clusters in active and silent domains, in growing limb cells, also involves differential association with surrounding sequences. In the developing forebrain, where the entire *HoxD* cluster is repressed, some of these long-range interactions with distant islands are observed too, yet in this case the islands display a distinct epigenetic signature, involving limited levels of H3K4me1 and no H3K27Ac [60]. This indicates that the transition between a ground-state or poised structure towards a fully active configuration involves only a partial reorganization of the locus micro-architecture, associated with chromatin modifications over the regulatory elements (figure 2*b*). Interestingly, such megabase-scale regions of chromatin interactions, or ‘topological domains’ are widespread in vertebrate genomes, and appear stable when comparing different cell lines [77], suggesting that ‘poised’ regulatory conformations are not restricted to *Hox* clusters.

However, when using chromosome conformation capture approaches, it is important to keep in mind a few methodological aspects. First, the data obtained give an average of the contacts established within the cellular population under scrutiny and hence may not reflect the real situation within one particular cell, at a particular time. While this approach does not tell about the dynamics of the interactions, the subsequent use of contact points as baits themselves, may at least give some hints about the complexity of the interactions (for example by documenting interactions amongst various enhancers sequences, rather than between enhancers and the target promoter). In any case, the cellular homogeneity of the sample and its physiological relevance must be carefully assessed beforehand to help interpret the interaction profiles.

Second, the cross-linking step will bring together DNA pieces that can be quite far from one another, provided they are engaged in large DNA–protein complexes. In this respect, the notion of ‘contact’ can be understood both as a direct and close interaction between two pieces of DNA, for example between an enhancer sequence and its target promoter *via* looping, but also as a more diffuse proximity, where enhancers could act as platforms to recruit factors such as to increase their concentration around particular genomic loci. In this context, investigating these dynamic patterns of contacts at the single cell level and at different stages of limb development should bring more insight into the establishment of this complex conformation. Interestingly, recent observations indicate that the frequencies of some of these interactions, as documented by FISH, display regional differences along the limb anterior–posterior axis [78].

Several of the regulatory islands, as defined by the interaction profile, could elicit a digit-specific transcriptional activation when isolated from the locus and tested in transgenic assays [60,68,79], further validating the 4C approach as a tool to isolate long-range acting enhancers. A genetic dissection of the 800 kb large genomic interval centromeric to *Hoxd13* indicated that these various elements contribute in a partially redundant fashion to the transcriptional activation of *Hoxd* genes ([60]; figure 2*c*). Such a dispersed enhancer system, somehow reminiscent of shadow enhancers described in *Drosophila* [80–82]*,* was referred to as a regulatory archipelago, i.e. a collection of regulatory islands, located nearby one another and all devoted to a collective function though with slightly different contributions of the diverse elements. Such a complex regulatory system may both ensure a robust transcriptional response of the target genes, and confer some regulatory flexibility during the development and evolution of distal limbs. Genetic alterations within this interval could indeed impact on the growth and patterning of digits, and thus contribute to the diversity of digit morphologies amongst various tetrapods.

## 6. Concluding remarks and outlook

The correspondence between the structural organization of *Hox* clusters and their transcriptional control in space and time has fascinated biologists for decades. While the underlying mechanisms remain to be fully elucidated, recent progress in deciphering the epigenetic status of these loci in developing embryos, as well as their three-dimensional organization in the nuclear space, have provided substantial information.

Future investigations will involve the identification of upstream factors and signals controlling the recruitment of chromatin-modifying activities to *Hox* loci, as well as the definition of their spatial organization during development. Considerable interest has been focused recently on the involvement of non-coding RNA or structural proteins, such as CTCF, in these processes [56,58,83,84]. For instance, CTCF binding sites are enriched at the boundaries of topological domains within the *HoxA* cluster, as well as at other genomic loci [77]. Yet, a critical impact of these various factors on *Hox* clusters regulation has not been confirmed by genetic analysis, so far [85,86]. It will also be critical to isolate the factors involved in the transcriptional activation of *Hox* genes, to understand the nature of the directionality of the ancestral process, during trunk extension.

Finally, the limb regulatory archipelago may be a paradigm to understand the emergence and evolution of such regulatory systems. Tetrapods indeed present a great variety of distal morphologies, in their limbs, and the analysis of the accompanying regulations will be informative in this context. Also, developing limbs are accessible structures, in the developing embryo, with rather well defined cell types and expression domains, unlike cultured cell systems, which may have little physiological relevance in this context, and where genetic approaches are not available.

Tracing back the emergence of novel regulatory landscapes in vertebrates may also bring some insights to our understanding of genome evolution. For instance, the acquisition of a late and distal phase of *Hoxd* gene activation in limbs was critical to the evolution of tetrapod digits, and this expression specificity does not seem to have a counterpart in fishes [87]. Likewise, the activation of *Hoxd* genes in developing external genitalia and digits follows highly similar patterns, which led to the hypothesis of a shared and ancestral regulation in both structures [22,88]. Addressing the regulatory potential of the syntenic regions of different species, as well as comparing the mechanisms controlling *Hox* clusters expression in different embryonic structures, should help reconstruct the evolutionary history of these regulations.

Similar concepts are likely to apply to other regulatory contexts. For instance, the involvement of gene deserts in long-range transcriptional control is not a specificity of *Hox* clusters, and regulatory elements were identified within gene deserts flanking other genes displaying intricate expression patterns [89–92]. The prevalence of regulatory archipelagos similar to that described at the *HoxD* locus nevertheless remains to be established and further efforts in mapping the spatial organization of genomes, combined with large-scale genetic approaches will be necessary.
